# Exclusive percutaneous peripheral veno-arterial ECMO with distal reperfusion of homolateral limb

**DOI:** 10.1186/1749-8090-10-S1-A260

**Published:** 2015-12-16

**Authors:** Mazzoni Lucia, Azmoun Alexandre, Ramadan Ramzi, Ghostine Saïd, Kloeckner Martin, Brenot Philippe, Fradi Mohamed, Nottin Rémi, Deleuze Philippe

**Affiliations:** 1 Adult Cardiac Surgery Department, Marie Lannelongue Hospital, Le Plessis Robinson, 92350 France

## Background/Introduction

ECMO is widely used through the world, but is still surrounded by important complications, most of them at the site of the surgical approach.

## Aims/Objectives

We describe and assess the effectiveness, the feasibility and the security of exclusive percutaneous peripheral veno-arterial ECMO including the distal reperfusion of the homolateral lower limb.

## Method

It is a monocentric, retrospective and observational study. Twenty nine patients (seventeen men, mean age 60,7 years) were implanted between July 2012 and March 2014. The indications for ECMO were acute phase of myocardial infarction (n = 8), post-cardiotomy cardiogenic shock (n = 12), acute myocarditis (n = 1), cardiac rhythm disorders (n = 1), bridge to cardiac transplantation or long-term circulatory support (n = 7). The insertion was performed in hybrid operating room. After puncture of the common femoral artery (CFA) the superficial femoral artery (SFA) was opacifiated. An anterograde 6F introducer was placed in the SFA under fluoroscopic control. The arterial and venous cannulae were then introduced through guide wires placed under fluoroscopic control. The distal perfusion of the homolateral lower limb was performed through a connection between the arterial cannulae and the introducer placed initially. Femoral arterial cannula was surgically removed at the end of the assistance to avoid vascular complications, the others by compression.

## Results

The mean duration of circulatory support was 9,5 days (from 1 to 52 days). Thirteen deaths occurred (45%). Bleeding of the groin required a haemostasis procedure in two cases only (5%). One external iliac artery dissection (2,5%) and two acute thrombosis of the oxygenator (one of which was reversible) (5%) occurred. Neither infection, nor lower limb acute ischemia occurred. A percutaneous atrioseptostomy was performed in catheterization room in eight patients who presented an acute pulmonary edema (21%), which all resumed.

## Discussion/Conclusion

These results suggest that the implantation of exclusive percutaneous peripheral veno-arterial ECMO is efficient, reproducible and minimally invasive. The pre-positioning of the distal reperfusion should be systematic. Surgical ablation of the arterial femoral cannula is necessary, but complications related to an initial surgical approach could be avoided during the time of assistance.

