# Correction to: Exclusive percutaneous peripheral veno-arterial ECMO with distal reperfusion of homolateral limb

**DOI:** 10.1186/s13019-018-0722-9

**Published:** 2018-05-10

**Authors:** Lucia Mazzoni, Alexandre Azmoun, Ramzi Ramadan, Saïd Ghostine, Martin Kloeckner, Philippe Brenot, Mohamed Fradi, Rémi Nottin, Philippe Deluze

**Affiliations:** Adult Cardiac Surgery Department, Marie Lannelongue Hospital, 92350 Le Plessis Robinson, France

## Correction

The original article [1] contains an error whereby all authors’ names are mistakenly inverted; this was an error mistakenly carried forward by the production team that handled this article, and thus was not the fault of the authors. As such, the correct configuration of the authors’ names can be viewed in this Correction article.
